# English language learning anxiety and academic burnout in Chinese freshmen: a chain mediating approach

**DOI:** 10.3389/fpsyg.2024.1340142

**Published:** 2024-06-04

**Authors:** Shuang Zheng, Junliang Zhang, Jingming Wang, Ruiqing Shen

**Affiliations:** Science and Technology College, Nanchang Hangkong University, Jiujiang, China

**Keywords:** learning anxiety, academic burnout, academic peer support, academic self-efficacy, freshmen

## Abstract

**Background:**

The university stage is critical for cultivating and enhancing students’ practical English proficiency, encompassing listening, speaking, reading, writing, and translation skills. Losing interest in English studies at this stage not only hampers the development of practical English competence but also has a negative impact on future employment and personal growth.

**Aims:**

This study aimed to explore to how English language learning anxiety (ELLA) affects academic burnout (AB) of freshman in China and explores the role of academic peer support (APS) and academic self-efficacy (ASE) in the relationship between the two.

**Methods:**

The study involved 1,355 college students who completed English Language Learning Anxiety Scale, the English Academic burnout Scale, Academic Peer Relationship Scale, the Academic Self-efficacy Scale.

**Results:**

AB was significantly impacted by ELLA. ELLA and AB were mediated by ASE. Another factor that acted as a mediator between ELLA and AB was APS. ELLA and AB were mediated by a chain reaction involving ASE and APS.

**Conclusion:**

The chain mediation model validated in this study, providing valuable insights into the effects of freshmen’s ELLA on AB in China, as well as practical insights into the prevention and intervention of ELLA and AB in other current college students.

## Introduction

Despite the extensive educational reforms in China over the years, contemporary Chinese college students commonly experience ELLA and English AB (Xu et al., 2022a; Liu et al., 2023). English AB is characterized by learners’ negative emotional experiences and low self-efficacy, resulting from emotional exhaustion and a lack of motivation during English learning (Xu et al., 2022b). This, in turn, adversely impacts the learning process. College students commonly experience English AB, manifested in low mood, reduced sense of achievement, and avoidance behavior, which is a prevalent negative psychological phenomenon (He et al., 2023). It not only endangers students’ physical and mental health (Yu et al., 2022), but also impacts their social interactions to the extent that they may experience burnout upon entering society (Wei et al., 2021). Given that burnout negatively impacts learners’ psychology, an increasing number of experts recognize it as a crucial factor that should not be overlooked, given its influence on learners’ effectiveness in learning. Furthermore, related studies indicate a negative correlation between AB and academic performance (March-Amengual et al., 2022). Many learners of foreign languages experience AB due to the inherent challenges in their learning, particularly because they lack immersion in a language-natural environment (Liu et al., 2021). Despite years of English teaching reforms and some achievements, AB is on the rise among Chinese college students (with a focus on non-English majors) each year. This study aims to comprehensively examine and analyze college students’ English AB, exploring the influencing variables, causes, and underlying mechanisms of this phenomenon.

## Theory and hypotheses

### ELLA and AB

Among the many factors affecting AB, unquestionably, one of the biggest issues is ELLA, which is also a significant predictor of AB (Lepp et al., 2014). ELLA is an emotional condition of tension in the student body and an expectation of a particular learning outcome (such as one that could jeopardize realistic or expected self-esteem and values) (Warr, 2000). Some students may exhibit a dry throat, tingling in the hands and feet, fatigue, avoidance of direct eye contact with the teacher, and avoidance of interaction with the teacher in the classroom (Von Worde, 2003; Han, 2006). In addition to having an impact on teenagers’ academic performance, ELLA can cause mental health issues, low self-esteem, and other psychological issues. A number of studies have proved that ELLA, as a significant predictor of learning burnout, is a major factor affecting learners’ performance and acquisition (Mahmoodi-Shahrebabaki, 2016), and it can be said that the greater the ELLA, the more serious the AB in English (Wang et al., 2023; Zhu et al., 2023). ELLA can appear at various points during the learning process or in various contexts, particularly among pupils who are exclusively exposed to the language in language classes. Students who score highly on ELLA are typically prone to negative emotional experiences such as nervousness, worry, fear, dread, and fear during the learning process, and they hold avoidance attitudes toward various activities in foreign language classes, etc. (Zhang and Yuan, 2004). These negative experiences and attitudes will undoubtedly lead to learning burnout. The rapid development of globalization makes the society’s demand for graduates’ English higher and higher, and ELLA becomes more and more serious, and the degree of burnout is also increasing. How to reduce college students’ ELLA and alleviate AB is an urgent issue at present. This study hypothesizes that ELLA will have a significant positive effect on AB (H1).

### APS as a mediator

Academic peer support is defined as a communicative process with positive meaning based on the interaction of both students, which is essentially a positive and supportive classroom climate from peers (Johnson et al., 1983). For language learners, this reciprocal APS is essential due to the language learning challenges they will encounter (Hartup, 1989). According to Huang et al. (2010), students who had greater peer support experienced less anxiety and performed better when learning a foreign language. Peer support as a category of social support research, and social support is an essential element affecting learning burnout. Guo and Niu (2017) study found APS positively predicts performance when learning a foreign language. In other words, more peer support in the learning process can reduce ELLA, show better academic performance, and will be less prone to learning burnout. Good peer academic support is conducive to creating a pleasant learning environment, which has a negative correlation with AB (Allen et al., 2021). In the course of learning English, students experience ELLA, physical and mental weariness, peer support to create a strong feeling of group identity, which leads to a pleasant emotional experience, a decrease in burnout, and an improvement in academic achievement. Krashen (1985) believes that language learners in the learning process, once the learning anxiety, learning will be hindered, and at this time, the support of academic peers to make the individual feel at ease, the enhancement of academic achievement will be reduce the degree of AB. Therefore, we hypothesized that APS acts as a mediator in the connection between ELLA and AB (H2).

### ASE as a mediator

Renowned psychologist Bandura (1977) initially proposed the concept of self-efficacy, which is an individual’s presumption and judgment of whether he or she can complete a certain task. ASE, on the other hand, refers to a person’s self-assurance in their capacity to exert control over academic effort and performance and ability to successfully complete that academic task (Liang, 2000). Students with high self-efficacy not only motivate individuals to learn intrinsically, but are also able to adapt quickly to our changing environment, resulting in increased academic engagement and reduced AB (Nielsen et al., 2017). Some studies have even suggested that self-efficacy developed in school can be an important predictor for coping Strategies (Samfira and Paloş, 2021). Strong senses of self-efficacy enable people to take charge of their education, finish tasks effectively, perform well academically, and significantly lessen AB (Allari et al., 2020). Students who possess strong self-efficacy assume greater accountability for their own education and, thus, show greater confidence in solving and completing challenging academic tasks. In turn, this greater sense of responsibility and self-efficacy may also contribute to mitigating reduced levels of AB (Rohmani and Andriani, 2021).

According to Bandura’s social cognitive learning theory, some scholars also define the subjective judgment of learners’ ability to complete English learning tasks as self-efficacy in English learning (Huang, 2008). When the learning task is difficult or challenging, students who has high self-efficacy show stronger confidence and courage to overcome difficulties, and hardly show negative emotions such as tension and anxiety; while students with low self-efficacy lack challenge and fear competition, over exaggerate the learning difficulties and lack of ability, lack of aggressiveness, and are prone to experience unpleasant feelings, including tension and anxiety, or even depression (Zhang et al., 2022). At the same time, it has been found that learners with high levels of ELLA must negatively affect their academic performance, which in turn affects their level of self-efficacy, which further affects the degree of AB (Hood et al., 2020). Some empirical studies have also shown that self-efficacy is significantly negatively correlated with ELLA and burnout, while ELLA is significantly positively correlated with burnout (Tingting, 2022). This suggests that students who have an elevated sense of self-efficacy appear to have stronger learning ability, stronger ability to face frustration, and relatively less learning anxiety and burnout, and their learning effectiveness will naturally be better, and vice versa, which is consistent with Bandura’s (1997) view on self-efficacy. In summary, we hypothesize that ASE acts as a mediator in the association between ELLA and AB (H3).

### Mediating role of APS and ASE

According to the above analysis, APS or ASE may be mediating variables of ELLA and AB. However, are APS and ASE themselves linked? In this study we will center on whether they are both a chain mediating variable of academic anxiety and AB. Bandura’s theory of self-efficacy proposes that emotional arousal, verbal persuasion, and an individual’s direct and indirect experiences are all important factors that influence an individual’s level of self-efficacy. Also, Gist and Mitchell (1992) three-dimensional model of self-efficacy states that external control factors (e.g., external support) are one of the important dimensions of cognitive processing of self-efficacy. APS can lead to more self-efficacy for individuals, as shown in a study that a positive social-emotional climate (peer support and teacher support) can lead to better self-efficacy as students perceive more external support and experience more positive social emotions (Kiefer et al., 2015). Some empirical studies have confirmed that APS not only promotes academic performance and emotional support, but also enhances individual self-efficacy (Abrams et al., 2022). At the same time, some researchers have found that a person’s perception of self-efficacy can be weakened by a lack of APS, which in turn generates negative emotions (Jia et al., 2023). In related literature, we can also find that APS positively predicts self-efficacy (Zou et al., 2023) and that ASE partially mediates the relationship between peer support and AB (Chen et al., 2016). Peer support also works by influencing students’ academic behavior, learning psychology, and academic performance through the influence of self-efficacy (Zimmer, 2000; Meunier and Roskam, 2009). Based on this, we hypothesized that peer support and ASE act as chain mediators between ELLA and AB (H4).

### Current research

Although numerous research have examined how ELLA affects AB (Kim et al., 2018; Yang and Zhai, 2022), few studies to date have specifically investigated the interplay between students’ APS and ASE. Specifically, how the association between ELLA and AB is influenced by both ASE and APS at the same time. This project will investigate ASE (an intrinsic factor) as well as APS (an extrinsic factor) to examine how both can simultaneously influence the relationship between ELLA and burnout. Given that ELLA and AB occur in the classroom, at the same time, ELLA is a frequent interaction and evaluation between learners and their peers (Jin and Dewaele, 2018). Thus, more important than other kinds of support are the roles that teachers and peers play. From the above analysis, we can see that an in-depth analysis of the mechanism and process of the influence of ELLA on AB among non-English majors has profound scientific significance and important application value. The scientific significance lies not only in revealing the potential influence mechanism of ELLA on AB, but also in revealing the role and status of self-efficacy and peer support in this influence process, thus laying a theoretical foundation for the construction of a predictive model of AB with high efficiency of ELLA; and the practical application value is also obvious, which has certain inspiration for the reform and development of the education of English college students.

**FIGURE 1:**
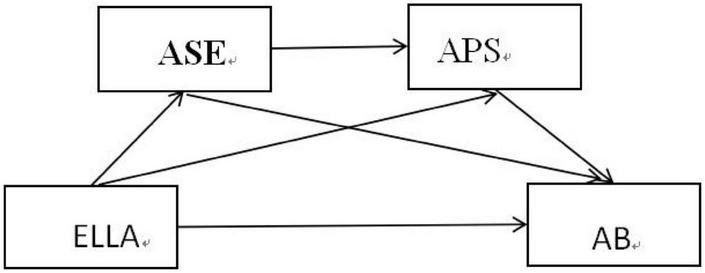
Graphical overview of the hypothesized effects.

In conclusion, this study intends to explore the influence mechanism of ELLA on college students’ AB, and construct a chain mediation model of ELLA on college students’ AB with APS and ASE as mediating variables. The hypothesis model is shown in Figure 1.

## Objects and methods

### Subjects

In this study, 1,355 college students not majoring in English were selected from three colleges and universities in Jiangxi Province of China by random sampling, and the questionnaires were distributed, completed and collected under the supervision of professionals. After excluding 63 invalid questionnaires, the valid questionnaires were 1292, with a validity rate of 95.35% and an average age of 18.83±0.27 years. The distribution of the sample was as follows: 934 boys (72.29%) and 358 girls (27.71%); 882 (68.27%) were from rural areas and 410 (31.73%) were from towns.

### Tools for research

#### Foreign Language Anxiety Scale (FLCAS)

In order to assess students’ ELLA, our study used the FLCAS developed by Horwitz et al. (1986). This scale, as a commonly used scale for testing ELLA, has been used extensively in the Chinese region and has proven its feasibility and validity. 22 items (e.g., “When I speak in English, I’m never completely sure of myself”)collectively comprise this scale. The scale items are categorized into four dimensions: communication anxiety, test anxiety, anxiety about negative evaluations, and other anxiety. The initial FLCAS was based on a five-point Likert scale and the scores were summed to give a total score. The greater the subject’s score, the more anxious they are. In this study, the Cronbach’s alpha for this scale was 0.96.

#### Academic Burnout Scale

The measure used in this study was derived from the English AB Scale for College Students compiled by Chinese scholars, which includes three dimensions: negative emotions, feelings of inefficiency and negative behaviors (Qizhi, 2011). The individuals were asked to score themselves on a 5-point Likert scale, and the scale included a total of 22 items (e.g., “I tend to get grumpy when I’m studying English”), with higher scores indicating a higher degree of English learning burnout. The Cronbach’s alpha for this scale in this study was 0.97.

#### Academic Peer Relationship Scale

In order to test students’ academic peer relationships, this study used the Student Peer Relationships Scale developed by Asher, which was adapted by Yali (2008), with a total of 16 items (e.g., “I make new friends easily at school”) consisting of three dimensions: welcoming, exclusionary, and isolation. The measure is rated on a 4-point scale ranging, with 10 items reverse scored, and the higher the general score for each subject, the better their peer relationships. The reliability of the scale was 0.94.

#### Academic Self-efficacy Scale

In this study, we used the Chinese version of the ASE Scale developed by Pintrich and de Groot (1990) and revised by Liang. Two dimensions were included: self-efficacy in learning ability and self-efficacy in learning behavior, with 11 entries in each dimension and a total of 22 entries (e.g., “In reading the book, I was able to understand what it was saying”). A five-point measuring scale was used, with higher scores representing higher ASE. The scale has strong reliability and validity and is capable for measuring college students’ ASE. The Cronbach’s alpha for the scale in this study was 0.95.

### Data processing

In this study, all analyses were performed using the Social Sciences Statistical Package (SPSS 26.0, IBM). First, Pearson correlation analysis was used to explore the relationship between ELLA, APS, ASE and AB. Secondly, PROCESS macro model 6 is used for mediation analysis. Indirect effects were estimated using 5,000 bootstrap samples, with 95% confidence intervals (CI) based on error-corrected estimates. If 95% CI does not include zero, the mediating effect is considered significant at *p* < 0.05 (Wen et al., 2012).

## Results

### Descriptive statistical analysis of the variables

Table 1 presents in detail the means, standard deviation and correlation coefficients of the main variables. Table 1 shows that all variables are significantly correlated with each other (*p* < 0.01), with APS (*r* = −0.38, *p* < 0.01), ASE (*r* = −0.36, *p* < 0.01) having a significant negative correlation with AB; and ELLA having a significant positive correlation with AB (*r* = 0.57, *p* < 0.01), and APS with ASE (*r* = 0.37, *p* < 0.01) were all significantly positively correlated.

**TABLE 1:** Means, standard deviations and correlation coefficients of the variables.

Variable	M	SD	1	2	3	4
1. ELLA	2.59	0.99	1			
2. ASE	3.00	0.79	−0.36**	1		
3. APS	0.80	7.29	−0.38**	0.37**	1	
4. AB	2.32	1.03	0.57**	−0.32**	−0.31**	1

**p < 0.01.

### Hypothesis test of chain mediation between APS and ASE

**FIGURE 2:**
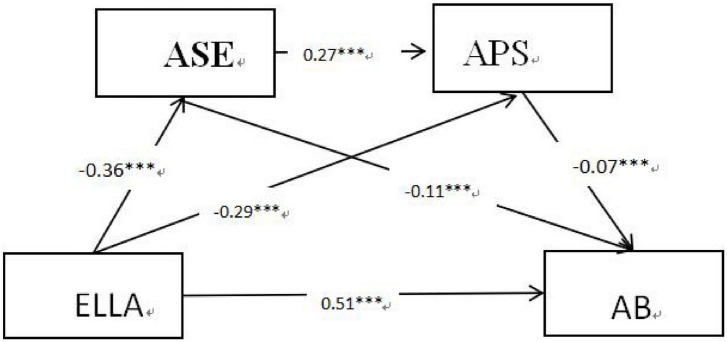
Chain mediation model of APS and ASE. ***p < 0.001.

Given the mediating role of APS and ASE between ELLA and AB, we use PROCESS 14. Macro model 6 was tested for mediation (Igartua and Hayes, 2021). According to the model (see Figure 2 and Table 2, ELLA has a considerable favorable influence on AB among college students.(β = 0.51, *p* < 0.001), and a significant negative effect on APS (β = −0.29, *p* < 0.001) and ASE (β = −0.36, *p* < 0.001); ASE negatively (β = −0.11, *p* < 0.001) affects AB and positively affects APS (β = 0.27, *p* < 0.001); APS negatively affects college students’ English AB (β = −0.07, *p* < 0.001), and ASE negatively affects college students’ AB (β = −0.13, *p* < 0.001).

**TABLE 2:** Regression analysis of chain mediation model.

Variable	ASE			APS			AB		
	**β**	***t***	**95%CI**	**β**	***t***	**95%CI**	**β**	***t***	**95%CI**
ELLA	−0.36	18.55***	[−0.40, −0.32]	−0.29	−14.35***	[−0.33, −0.21]	0.51	−26.81***	[0.47, 0.55]
ASE				0.27	13.26***	[0.23, 0.30]	−0.11	−5.61***	[−0.14, −0.07]
*APS*							−0.07	−3.86***	[−0.11, −0.04]
*R* ^2^	0.13			0.21			0.35***		
*F*	343.95***			298.89***			407.09***		

***p < 0.001.

The mediating effect values are were tested as follows (Table 3): The results showed that in the pathway of “ELLA → ASE → AB”, the confidence interval of APS was 95% CI [0.02, 0.06], which indicated that the partial mediating effect of APS in ELLA and AB had been tested, and H2 was established; In the path of “ELLA → APS → AB”, the confidence interval of ASE was 95% CI [0.01, 0.03], which indicated that the partial mediating effect of ASE in the paths of ELLA and AB was tested, and H3 was established; In the pathway of “ELLA → ASE → APS → AB”, the confidence interval of APS and ASE was 95% CI [0.00, 0.01], which indicated that the chain mediating effect of APS and ASE in ELLA and AB was tested, and H4 was established.

**TABLE 3:** Chain mediation effect analysis.

Effect	Pathways	Effect	Boot SE	BootLLCI	BootULCI
Direct effect	ELLA → AB	0.07	0.03	0.04	0.12
Indirect effect	ELLA → ASE → AB	0.04	0.02	0.02	0.06
	ELLA → APS → AB	0.02	0.01	0.01	0.03
	ELLA → ASE → APS → AB	0.01	0.004	0.00	0.01

***p < 0.001, **p < 0.01, *p < 0.05.

## Discussion

### The influence of ELLA on AB

According to this study’s findings, ELLA significantly reduces AB; that is, college students’ levels of AB increase with their ELLA, which is in line with other research findings (Xu et al., 2022b). Previous studies have shown that ELLA affects an individual’s attention, memory, and ability to retrieve (Moran, 2016), increases students’ learning difficulties, leads to a reduction in academic achievement, and in severe cases, leads to a loss of interest in learning (Shen, 2021). Of course, studies have also concluded that moderate anxiety can be a motivation for English learning and is beneficial, but excessive ELLA can have negative effects (Romano et al., 2020). Therefore for English language learners, the necessary ELLA is a normal reaction. Students under prolonged high ELLA are prone to self-doubt and negative effects on ELLA and learning emotions (e.g., AB) (Iqbal et al., 2015).

### Mediating role of APS

This study examined the mediating effect of APS between ELLA and AB, and the findings demonstrated that the connection between ELLA and AB was mediated by APS. This suggests that freshmen’s ELLA can influence AB through peer support. The model of social support buffering suggests that regardless of how much or how little social support an individual receives, it has a gainful effect on his or her mental health (Cohen and Wills, 1985). Previous studies have concluded that APS is strongly and adversely associated with ELLA, i.e., the more support received, the less anxiety is felt (Shaohua et al., 2018). Anxiety in English learning will inevitably affect freshmen’s commitment to English learning, and the beneficial influence of peer support will reduce the degree of individual ELLA, which in turn will reduce the possibility of English AB. If freshmen develop AB in English learning, they will feel physically and mentally exhausted, their interest in learning decreases, and then they will feel more anxious about English learning (Schaufeli et al., 2002). However, if they can get more peer support in the process of English learning, their learning burnout will decrease, their ELLA will decrease, and their interest in learning engagement will increase.

### The role of ASE as a mediator

In the analysis of mediating effects, we can also find that ASE plays a mediating role between ELLA and AB. This suggests that although freshmen’s ELLA inevitably has a direct impact on English learning burnout, ASE can play a role in alleviating AB and enhancing learning motivation. Additionally, in line with earlier studies, this study discovered that a person’s degree of AB decreased with increasing self-efficacy (Yang and Farn, 2005). Higher self-efficacy people are better equipped to use constructive coping mechanisms to deal with challenges, which is projected onto academic problems as being able to awaken the individual’s motivation to learn (Liem et al., 2007). Although ELLA affects freshmen’s AB and even self-efficacy, the emotional arousal function of self-efficacy will enable individuals to suppress their ELLA (Dogan, 2015), which will ultimately increase the desire to learn and reduce the level of AB in English.

### Chain mediation of APS and ASE

In addition, it was found that freshmen’s ELLA can also have an effect on English AB through APS and ASE. Also this study pointed out that APS and ASE were significantly positively correlated and positively influenced, which is in line with the study done by Chong et al. (2018). In the process of English learning, the more peer support freshmen receive, the more positive self-evaluations they have, the higher their individual self-efficacy is, and the less chance they have of AB. According to Connel et al.’s (1995) theoretical model of self-processing, APS increases academic engagement, and it is also believed that students’ self-efficacy for academics increases when they receive support and assistance from others . Peer support alters an individual’s self-efficacy through the individual’s prior achievement performance, alternative experiences, verbal persuasion, and psychological states (Krause et al., 2003; Wijaya et al., 2022). Positive peer evaluations, successful experiences, and positive psychological states can motivate individuals to maintain a good psychological state, enhance self-efficacy, increase self-confidence in learning, and alleviate AB. According to this study, in order to solve the issue of freshmen’s ELLA, it is not only necessary to pay attention to APS, enhance the learning environment, and provide external support for academic growth, but also to stimulate the learners’ own learning morale and enhance their self-efficacy, so as to eliminate AB fundamentally.

### Theoretical and practical implications

Drawing on previous research, this study employs the chain mediation model to examine the relationship between ELLA and AB among first-year students. The findings bear considerable practical implications for the English language acquisition and learning adjustment of first-year students in China. They also serve as a benchmark for improving teaching and learning standards in higher education in China. Previous research has focused on ELLA and AB in elementary and secondary education. The English language acquisition of college students, especially first-year students, has not received sufficient attention. The relationship between AB and fundamental ELLA, accounting for two variables in this study, provides a crucial theoretical foundation for enhancing freshmen’s English learning capacity.

### Shortcomings and prospects of this study

First, the sample selection has limitations, with a higher number of male students than female students in the subjects. Therefore, future studies should broaden the subject selection to improve sample representativeness, ensuring more objective and accurate results. Second, this study is cross-sectional, lacking causal inference. Future research could employ a longitudinal tracking study to address this limitation. Moreover, numerous factors influence ELL and AB. Future research should explore additional mediating variables in the intrinsic influence mechanism for in-depth analysis and exploration. Finally, the study’s sample was limited to China, considering variations in cultural and educational backgrounds. Consequently, the reproducibility and generalizability of findings may be somewhat limited. Therefore, future studies should expand the sample to collect data for testing these findings.

## Conclusion

This study investigated the correlation between freshmen’s ELLA and AB, as well as the association between APS and ASE:

First, significant correlations were found among ELLA, APS, ASE, and AB. Second, APS and ASE served as mediators in the relationship between ELLA and AB. Third, APS and ASE played a chain mediating role between ELLA and AB.

In summary, ELLA can influence AB through the pathways of APS and ASE. This necessitates English teaching staff to prioritize ELLA and offer suitable assistance. Additionally, attention should be given to fostering APS through the establishment of relevant groups, fostering a positive learning atmosphere. Finally, providing students with positive feedback is crucial for boosting morale, strengthening self-efficacy in learning, and mitigating AB.

## Data availability statement

The raw data supporting the conclusions of this article will be made available by the authors, without undue reservation.

## Ethics statement

The studies involving humans were approved by the Science and Technology College, Nanchang Hangkong University, Jiujiang, China. The studies were carried out in compliance with institutional guidelines and local laws. To take part in this study, the participants provided written informed consent to participate in this study.

## Author contributions

SZ: Conceptualization, Data curation, Formal analysis, Validation, Writing – original draft. JZ: Conceptualization, Data curation, Writing – original draft. JW: Investigation, Validation, Writing – review and editing. RS: Visualization, Writing – review and editing.

## References

[B1] Abrams M. P. Salzman J. Espina Rey A. Daly K. (2022). Impact of providing peer support on medical students’ empathy, self-efficacy, and mental health stigma. *Int. J. Environ. Res. Public Health* 19(9):5135. 10.3390/ijerph19095135 35564535 PMC9099875

[B2] Allari R. S. Atout M. Hasan A. A. (2020). The value of caring behavior and its impact on students’ self-efficacy: perceptions of undergraduate nursing students. *Nurs. Forum.* 55 259–266. 31950519 10.1111/nuf.12424

[B3] Allen H. K. Barrall A. L. Vincent K. B. Arria A. M. (2021). Stress and burnout among graduate students: moderation by sleep duration and quality. *Int. J Behav. Med.* 28 21–28. 10.1007/s12529-020-09867-8 32124246 PMC7483179

[B4] Bandura A. (1977). Self-efficacy: toward a unifying theory of behavioral change. *Psychol. Rev.* 84 191–215. 10.1037//0033-295x.84.2.191 847061

[B5] Bandura A. (1997). *A Self-Efficacy: the Exercise of Control.* New York, NY: Freeman and company.

[B6] Chen W. Zhao S. Han H. Wei W. Zhang J. (2016). The relationship between social support, academic self-efficacy and academic burnout among high school students. *Teach. Manag.* 2 70–73. 10.3390/ijerph20126070 37372657 PMC10298565

[B7] Chong W. H. Liem G. A. Huan V. S. Kit P. Y. Ang R. P. (2018). Student perceptions of self - efficacy and teacher support for learning in fostering youth competencies: roles of affective and cognitive Engagement. *J. Adolesc.* 68 1–11. 10.1016/j.adolescence.2018.07.002 29986166

[B8] Cohen S. Wills T. A. (1985). Stress social support and the buffering hypothesis. *Psychol. Bull.* 98 310–357. 3901065

[B9] Connel J. P. Halpern-Felshsher B. L. Clifford E. Crichlow W. Usinger P. (1995). Hanging in there: behavioiral, psychological, and contextual factors affecting whether African American adolescents stay in high school. *J. Adolesc. Res.* 10 41–63.

[B10] Dogan U. (2015). Student engagement, academic self- efficacy, and academic motivation as predictors of academic performance. *Anthropologist* 20 553–561.

[B11] Gist M. E. Mitchell T. R. (1992). Self-efficacy: a theoretical analysis of its determinants and malleability. *Acad. Manag. Rev.* 17(2), 183–211. 10.2307/258770

[B12] Guo J. Niu R. (2017). A study of teacher-student support in English learning and its relationship with academic performance. *For. Lang. Transl.* 24 67–71.

[B13] Han Z. (2006). *Second Language Anxiety and Coping Strategies. Journal of Shandong Province Youth Management Cadre College*. 2, 128–130.

[B14] Hartup W. W. (1989). Social relationships and their development significance. *Am. Psychol.* 44 120–126.

[B15] He L. Yuan X. Chen Q. Wang X. (2023). Intrusive rumination and academic burnout among adolescents in ethnic minority areas of China during the COVID-19 pandemic: PTSS as mediator and cognitive reappraisal as moderator. *BMC Public Health* 23:2201. 10.1186/s12889-023-17133-1 37940905 PMC10634029

[B16] Hood S. Nancy B. Djerdjian N. Farr M. Gerrits R. J. Lawford H. (2020). Some believe, not all achieve: the role of active learning practices in anxiety and academic self-efficacy in first-generation college students â. *J. Microbiol. Biol. Educ.* 21. 10.1128/jmbe.v21i1.2075 32313594 PMC7148146

[B17] Horwitz E. K. Horwitz M. B. Cope J. A. (1986). Foreign language classroom anxiety. *Modern Lang. J.* 70 125–132.

[B18] Huang R. (2008). Exploring the self-efficacy of college students’ English learning. *J. Inner Mongolia Agric. Univer.* 10 48–49.

[B19] Huang S. Eslami Z. Hu R.-J. S. (2010). The relationship between teacher and peer support and english-language learners’anxiety. *Engl. Lang. Teach.* 3.

[B20] Igartua J. J. Hayes A. F. (2021). Mediation, moderation, and conditional process analysis: concepts, computations, and some common confusions. *Spanish J. Psychol.* 24:e49. 10.1017/SJP.2021.46 35923144

[B21] Iqbal S. Gupta S. Venkatarao E. (2015). Stress, anxiety & depression among medical undergraduate students & their socio-demographic correlates. *Indian J. Med. Res.* 141 354–357. 10.4103/0971-5916.156571 25963497 PMC4442334

[B22] Jia J. Ma Y. Xu S. Zheng J. Ma X. Zhang Y. (2023). Effect of academic self-efficacy on test anxiety of higher vocational college students: the chain mediating effect. *Psychol. Res. Behav. Manag.* 16 2417–2424. 10.2147/PRBM.S413382 37426390 PMC10327914

[B23] Jin Y. X. Dewaele J.-M. (2018). The effect of positive orientation and perceived social support on foreign language classroom anxiety. *System* 74 149–157.

[B24] Johnson D. W. Roger J. Douglas A. (1983). Social interdependence and classroom climate. *J. Psychol.* 114 135–142. 10.1080/00223980.1983.9915406

[B25] Kiefer S. M. Alley K. M. Ellerbrock C. R. (2015). Teacher and peer support for young adolescents’ motivation, engagement, and school belonging. *RMLE Online* 38(8), 1–18. 10.1080/19404476.2015.11641184

[B26] Kim B. Jee S. Lee J. An S. Lee S. M. (2018). Relationships between social support and student burnout: a meta-analytic approach. *Stress Health* 34 127–134. 10.1002/smi.2771 28639354

[B27] Krashen S. (1985). *The Input Hypothesis: Issues and Implications.* New York: Longman.

[B28] Krause K. L. Mcinnis C. Welle C. (2003). Out-of-class engagement in undergraduate learning communities: the role and nature of peer interactions. *For Countries* 8.9, 259–261.

[B29] Lepp A. Barkley J. E. Karpinski A. C. (2014). The relationship between cell phone use, academic performance, anxiety, and satisfaction with Life in college students. *Comput. Hum. Behav.* 31 343–350. 10.1016/j.chb.2013.10.049

[B30] Liang Y. (2000). *A Study of College Students’ Achievement Goals, Attributional Styles and Academic Self-Efficacy.* Hubei: Huazhong Normal University.

[B31] Liem A. D. Lau S. Nie Y. (2007). The role of self-efficacy, task value, and achievement goals in predicting learning strategies, task disengagement, peer relationship, and achievement outcome. *Contemp. Educ. Psychol.* 33 486–512.

[B32] Liu C. He J. Ding C. Fan X. Hwang G.-J. Zhang Y. (2021). Self-oriented learning perfectionism and English learning burnout among EFL learners using mobile applications: the mediating roles of English learning anxiety and grit. *Learn. Individ. Dif.* 88:102011. 10.1016/j.lindif.2021.102011

[B33] Liu H. Zhang Z. Chi C. Tao X. Zhang M. (2023). Exploring the impact of the COVID-19 pandemic on academic burnout among nursing college students in China: a web-based survey. *Med. Sci. Monitor Basic Res.* 29:e940997. 10.12659/MSMBR.940997 37963323 PMC10624139

[B34] Mahmoodi-Shahrebabaki M. (2016). The effect of perfectionism on burnout among english language teachers: the mediating role of anxiety. *Teach. Teach.* 23 91–105. 10.1080/13540602.2016.1203776

[B35] March-Amengual J. M. Cambra Badii I. Casas-Baroy J. C. Altarriba C. Comella Company A. Pujol-Farriols R. (2022). Psychological distress, burnout, and academic performance in first year college students. *Int. J. Environ. Res Public Health* 19:3356. 10.3390/ijerph19063356 35329044 PMC8953100

[B36] Meunier J. C. Roskam I. (2009). Self-Efficacy beliefs amongst parents of young children: validation of a self-report measure. *J. Child Fam. Study* 18 495–511.

[B37] Moran T. P. (2016). Anxiety and working memory capacity: a meta-analysis and narrative review. *Psychol. Bull.* 142 831–864. 10.1037/bul0000051 26963369

[B38] Nielsen T. Makransky G. Vang M. L. Dammeyer J. (2017). How specific is specific self-efficacy?: a construct validity study using Rasch measurement models. *Stud. Educ. Eval.* 53 87–97.

[B39] Pintrich P. R. de Groot E. V. (1990). Motivational and self-regulated learning components of classroom academic performance. *J. Educ. Psychol.* 82 33–40. 10.1037/0022-0663.82.1.33

[B40] Qizhi Z. (2011). Research on college students’ english learning burnout. *J. Chongqing Univers. Technol.* 25 130–134.

[B41] Rohmani N. Andriani R. (2021). Correlation between academic self-efficacy and burnout originating from distance learning among nursing students in Indonesia during the coronavirus disease 2019 pandemic. *J. Educ. Eval. Health Professions* 18:9. 10.3352/jeehp.2021.18.9 33971088 PMC8187029

[B42] Romano L. Tang X. Hietajarvi L. Salmela-Aro K. Fiorilli C. (2020). Students’ trait emotional intelligence and perceived teacher emotional support in preventing burnout: the moderating role of academic anxiety. *Int. J. Environ. Res. Public Health* 17:4771. 10.3390/ijerph17134771 32630744 PMC7369914

[B43] Samfira E. M. Paloş R. (2021). Teachers’ personality, perfectionism, and self-efficacy as predictors for coping strategies based on personal resources. *Front. Psychol.* 12:751930. 10.3389/fpsyg.2021.751930 34795619 PMC8593193

[B44] Schaufeli W. B. Martinez I. M. Pinto A. M. Salanova M. Bakker A. B. (2002). Burnout and engagement in university students:a cross-national study. *J. Cross Cult. Psychol.* 33 464–481.

[B45] Shaohua J. Su C. Wang T. (2018). Direct and indirect relationships between social support and quality of life among rural secondary school students in Sichuan province: the mediating role of Internet addiction and anxiety. *Health Soc. Behav.* 18 3364–3392.

[B46] Shen J. (2021). A review of the effectiveness of foreign language enjoyment and foreign language classroom anxiety on learners’ engagement and attainment. *Front. Psychol.* 12:749284. 10.3389/fpsyg.2021.749284 34552544 PMC8450351

[B47] Tingting Z. (2022). An empirical study on the relationship between college students’ self-efficacy, foreign language learning anxiety and learning burnout. *J. Southwest Jiaotong Univers.* 23(6), 55–64.

[B48] Von Worde R. (2003). Students’perspectives on foreign language anxiety. *Inquiry* 8:n1. 10.1007/s10639-022-11155-9 35756361 PMC9206888

[B49] Wang H. Wang Y. Li S. (2023). Unpacking the relationships between emotions and achievement of EFL learners in China: engagement as a mediator. *Front. Psychol.* 14:1098916. 10.3389/fpsyg.2023.1098916 36891212 PMC9986489

[B50] Warr P. (2000). Downing J. learning strategies, learning anxiety and knowledge acquisition. *Br. J. Psychol.* 91 311–333. 10.1348/000712600161853 10958577

[B51] Wei H. Dorn A. Hutto H. Webb Corbett R. Haberstroh A. Larson K. (2021). Impacts of nursing student burnout on psychological well-being and academic achievement. *J. Nursing Educ.* 60 369–376. 10.3928/01484834-20210616-02 34232812

[B52] Wen Z. Liu H. Hou J. (2012). *Analysis of Moderating and Mediating Effects.* Beijing: Educational Science Press.

[B53] Wijaya T. T. Rahmadi I. F. Chotimah S. Jailani J. Wutsqa D. U. (2022). A case study of factors that affect secondary school mathematics achievement: teacher-parent support, stress levels, and students’ well-being. *Int. J. Environ. Res. Public Health* 19:16247. 10.3390/ijerph192316247 36498321 PMC9737574

[B54] Xu L. Wang H. Chen J. Zhang Y. Huang Z. Yu C. (2022a). English learning stress, self-efficacy, and burnout among undergraduate students: the moderating effect of mindfulness and gender. *Int. J. Environ. Res. Public Health* 19:15819. 10.3390/ijerph192315819 36497890 PMC9739790

[B55] Xu L. Wang Z. Tao Z. Yu C. (2022b). English-learning stress and performance in Chinese college students: a serial mediation model of academic anxiety and academic burnout and the protective effect of grit. *Front. Psychol.* 13:1032675. 10.3389/fpsyg.2022.1032675 36533059 PMC9749891

[B56] Yali Z. (2008). *A Study Related to Middle School Students’ Self-Concept and School Adjustment.* Shangxi: Northwest University

[B57] Yang H. J. Farn C. K. (2005). An investigation the factors affecting MIS student burnout in technical -vocational college. *Comput. Hum. Behav.* 21 917–932.

[B58] Yang M. Zhai Y. (2022). Exploring the relationship between conception of language learning and foreign language learning burnout: an empirical study among university students. *Int. J. Cogn. Informatics Nat. Intell.* 16 1–12.

[B59] Yu X. Wang Y. Liu F. (2022). Language learning motivation and burnout among english as a foreign language undergraduates: the moderating role of maladaptive emotion regulation strategies. *Front. Psychol.* 13:808118. 10.3389/fpsyg.2022.808118 35185728 PMC8854853

[B60] Zhang R. Yuan L. (2004). A study of the relationship between college students’ foreign language anxiety, self-efficacy and foreign language performance. *Psychol. Dev. Educ.* 3 56–61.

[B61] Zhang W. Xiong S. Zheng Y. Wu J. (2022). Response efficacy and self-efficacy mediated the relationship between perceived threat and psychic anxiety among college students in the early stage of the COVID-19 pandemic. *Int. J. Environ. Res. Public Health* 19:2832. 10.3390/ijerph19052832 35270528 PMC8910033

[B62] Zhu P. Xu T. Xu H. Ji Q. Wang W. Qian M. (2023). Relationship between anxiety, depression and learning burnout of nursing undergraduates after the COVID-19 epidemic: the mediating role of academic self-efficacy. *Int. J. Environ. Res. Public Health* 20:4194. 10.3390/ijerph20054194 36901200 PMC10002455

[B63] Zimmer B. J. (2000). Self-efficacy: an essential motive to learn. *Contemp. Educ. Psychol.* 25 82–91 10620383 10.1006/ceps.1999.1016

[B64] Zou Y. Liu S. Guo S. Zhao Q. Cai Y. (2023). Peer support and exercise adherence in adolescents: the chain-mediated effects of self-efficacy and self-regulation. *Children* 10:401. 10.3390/children10020401 36832530 PMC9955246

